# Malposition of a Port Catheter in the Azygos Vein: Endovascular Repositioning Using a Long Loop Snare Technique

**DOI:** 10.1002/ccd.70618

**Published:** 2026-04-14

**Authors:** Axel Rentzsch, Annabelle Wagner, Jochen Pfeifer, Hashim Abdul‐Khaliq

**Affiliations:** ^1^ Department of Pediatric Cardiology Saarland University Medical Center Homburg Germany; ^2^ Department of Pediatric Oncology and Hematology Saarland University Medical Center Homburg Germany

## Abstract

Port catheters provide a reliable, long‐term venous access option in children for repeated administration of medications or parenteral nutrition. A cardiac catheterization procedure was performed in a 7‐year‐old girl in whom lateral chest radiography revealed posterior deviation suggestive of azygos vein malposition. To avoid surgical replacement, a long loop snare technique was employed to safely withdraw the catheter into the superior vena cava with restoration of catheter function. This case highlights that endovascular long loop snare repositioning offers a feasible, minimally invasive alternative to surgical intervention for rare port catheter malpositions in pediatric patients.

## 1 Case Report

**Figure 1:**
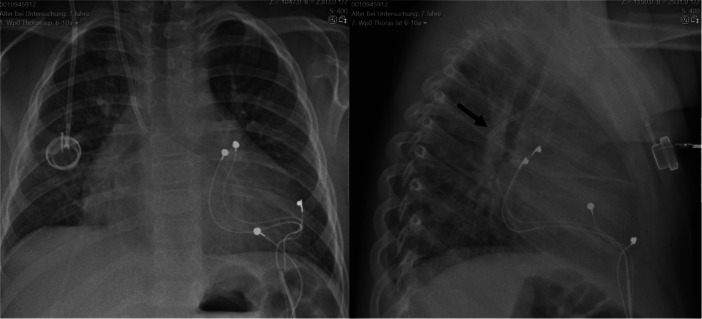
In the posteroanterior view, the catheter appeared to be correctly positioned within the superior vena cava; the lateral view revealed posterior deviation of the catheter tip (arrow).

We report on a 7‐year‐old girl with CHARGE syndrome and multiple associated comorbidities and prior interventions, including developmental delay, structural epilepsy, choanal atresia, dysphagia, transposition of an aberrant right subclavian artery, sick sinus syndrome with subsequent pacemaker implantation, and several urological surgeries for high‐grade vesicoureteral reflux. Additional history included malrotation of the small bowel with laparotomies and percutaneous endoscopic gastrostomy (PEG) placement. Enteral nutrition was largely insufficient, and the patient was therefore predominantly dependent on parenteral nutrition via a central access. The patient had multiple central lines before with several complications, such as central line associated infections, catheter malfunctions, dislocation and venous thrombosis. For the latter, treatment with rivaroxaban was ongoing. The current port catheter, the second implanted in this patient, had been placed 4 weeks prior to the intervention described here. Aspiration of blood through the catheter was possible only once following implantation. The present hospital admission was prompted by high fever, elevated inflammatory markers, and positive blood cultures. Targeted antibiotic therapy was initiated according to culture sensitivities. Transthoracic echocardiography was performed to assess the port catheter tip for possible infected thrombi or vegetations, but did not clearly identify the catheter within the superior vena cava, which in turn led to further evaluation by a two‐view chest radiography. While the frontal view suggested correct positioning, the lateral projection demonstrated posterior deviation of the catheter tip, raising concern for malposition (Figure 1).

Cardiac catheterization performed the following day confirmed malposition of the port catheter by retrograde opacification of the azygos vein extending into the inferior vena cava after contrast injection through the device (Movie S1). The vein was patent but demonstrated delayed contrast clearance. To avoid explantation and reinsertion of the port system, an interventional approach was chosen. Therefore two femoral venous sheaths were placed. Using a 5 F left internal mammary artery catheter (LIMA), the superior vena cava was first demonstrated to be unobstructed. The port catheter was captured with the LIMA catheter, then a 0.035‐inch guidewire was introduced through the LIMA catheter. Then a snare catheter with a 10 mm retrieval loop was advanced via the second sheath and captured the guidewire. Care was taken to establish a catheter‐on‐catheter configuration without contact to the guidewire to avoid direct traction on the wire and possible damage of the fragile port catheter, while ensuring stable countertraction for repositioning (Movie S2). Once the guidewire was secured on the proximal end of the LIMA catheter, gentle, coordinated traction on both catheters allowed the port catheter to be withdrawn into the superior vena cava with minimal and steady force (Movie S3). Final position was confirmed angiographically, and free blood aspiration through the catheter was restored. The patient was discharged 6 days later in good condition.

## 2 Discussion

Port catheters provide a reliable, long‐term venous access option in children for repeated administration of medications or parenteral nutrition. Complications occur in approximately 10% of cases and include thrombosis, reservoir protrusion, pneumothorax, infection, or catheter malposition [1]. Malposition into the azygos vein may lead to severe complications such as veno‐bronchial or broncho‐esophageal fistula formation [2]. In addition, spontaneous migration of a correctly positioned port catheter into the azygos vein, with subsequent development of a tracheo‐azygos fistula, has also been reported [3]. Rare variants such as absence of the azygos vein with hemiazygos continuation draining into the left brachiocephalic vein, or via a prominent left superior intercostal vein into a persistent left superior vena cava, have been described and may theoretically predispose to malposition, particularly with left‐sided venous access [4]. A persistent left superior vena cava may drain via an unroofed coronary sinus into the left atrium, creating a functional right‐to‐left shunt.

To our knowledge, catheter‐based interventional repositioning of a malpositioned port catheter has not previously been reported in children. This unconventional approach aimed to correct the malposition and eliminate the need for surgical replacement. The present intervention demonstrates the creation of a long loop, a technique described for similar procedures such as retrieval of catheter fragments [5, 6]. Previous reports have noted that a pigtail catheter alone may not provide sufficient traction to reposition a dislodged port catheter, particularly in the presence of vessel thrombosis. In our case, the relatively easy mobilization was likely facilitated by the short duration of malposition before intervention and by the patient's ongoing anticoagulation therapy.

This case highlights four diagnostic and therapeutic lessons.

First, lack of ultrasound visualization of a central venous catheter or its tip should prompt further imaging.

Second, relying solely on the posteroanterior projection in chest radiography may be insufficient, as azygos vein malposition could otherwise be overlooked.

Third, malposition into the azygos vein can be corrected by a long loop snare technique, thereby avoiding surgical replacement of the port system.

Fourth, when anticoagulation is indicated due to a history of catheter‐associated thrombosis, it may help limit thrombotic burden and facilitate interventional management in the event of catheter malposition.

## Consent

Written informed consent was obtained from the patient's parents for publication of this case report.

## Conflicts of Interest

The authors declare no conflicts of interest.

## Supporting information

Supporting Movie 1:

Supporting Movie 2:

Supporting Movie 3:

## Data Availability

The data that support the findings of this study are available from the corresponding author upon reasonable request.
